# Influence of Two Exercise Programs on Heart Rate Variability, Body Temperature, Central Nervous System Fatigue, and Cortical Arousal after a Heart Attack

**DOI:** 10.3390/ijerph20010199

**Published:** 2022-12-23

**Authors:** Catarina Gonçalves, Jose A. Parraca, Jorge Bravo, Ana Abreu, João Pais, Armando Raimundo, Vicente Javier Clemente-Suárez

**Affiliations:** 1 Departamento de Desporto e Saúde, Escola de Saúde e Desenvolvimento Humano, Universidade de Évora, 7004-516 Évora, Portugal; 2 Comprehensive Health Research Centre (CHRC), Universidade de Évora, 7004-516 Évora, Portugal; 3 Hospital de Santa Maria, 1649-028 Lisbon, Portugal; 4 Hospital Espírito Santo, 7000-811 Évora, Portugal; 5 Faculty of Sports Sciences, Universidad Europea de Madrid, 28670 Madrid, Spain

**Keywords:** cardiovascular diseases, heart rate variability, thermography, central nervous system fatigue, prognosis

## Abstract

Cardiovascular diseases (CVD) are the leading cause of death globally. Cardiac rehabilitation (CR) programs’ benefits are overall consensual; however, during exercise, progressive physiological effects have not been studied yet in cardiac patients. Our study aims to analyze physiological parameters of thermography, heart rate variability (HRV), blood pressure, central nervous system (CNS) fatigue, and cortical arousal in heart attack patients (HAP) who belong to CR programs of High-Intensity Interval Training (HIIT) and Moderate-intensity Continuous Training (MICT) compared to healthy participants. In this case control study, two HAP patients (both male, age 35 and 48, respectively) and two healthy people (both male, age 38 and 46, respectively) were randomly assigned in a 1:1:1:1 allocation ratio to one of four groups: cardiac MICT, cardiac HIIT, control MICT, and control HIIT. The HIIT at ≈85–95% of peak heart rate (HR) was followed by a one-minute recovery interval at 40% peakHR, and MICT at ≈70–75% of peakHR. Outcome measurements included thermography, HRV, blood pressure, CNS fatigue, and cortical arousal; The HAP presents more than twice the CNS fatigue in MICT than control participants, but HIIT has almost the same CNS fatigue in HAP and control. In addition, both of the HAP groups presented higher temperatures in the chest. The HIIT protocol showed better physiological responses during exercise, compared to MICT in HAP.

## 1. Introduction

According to World Health Organization (WHO) [1], cardiovascular diseases (CVD) are the number one cause of death globally. An estimated 17.9 million people died from CVD in 2019, representing 32% of all global deaths worldwide. Of these deaths, 85% were due to heart attack and stroke [1]. In 2019, there were 3.9 million deaths resulting from CVD in Europe, which corresponded to 45% of all deaths, considerably higher than the second most prevalent cause of death, cancer [2]. Furthermore, out of the 17 million premature deaths (under the age of 70) due to noncommunicable diseases in 2019, 38% were caused by CVD [1]. 

Cardiac rehabilitation (CR) is a multidisciplinary process for patients recovering after an acute cardiac event or chronic cardiovascular disease that reduces mortality and morbidity and improves the quality of life [3]. CR is the gold standard treatment for excellent recovery, not only physical but also mental and social after a cardiac episode so that their inclusion in daily life can be as normalized as possible; however, there is poor adherence to these types of programs, which could condition the recovery of patients [4], being that only 10% of patients with a CR indication attend these types of programs [5]. Two types of training are currently used in CR programs. Moderate-intensity continuous training (MICT) is routinely prescribed for cardiac patients in CR. Typically, the upper limit of intensity that is prescribed during the early stages of phase II cardiac rehab is 60–70% of heart rate reserve. This exercise intensity is performed continuously for 10–30 min, depending on endurance and as tolerated by the patient [6]. High-intensity interval training (HIIT) has been used as an effective type of training in healthy adults for many years. However, routine implementation of HIIT into CR programs for higher-risk cardiac patients has yet to be established. Recent clinical studies [7,8,9] have implemented HIIT into CR programs. The HIIT program allows patients to work at a higher intensity for two to three minutes, while alternating with recovery intervals at a moderate intensity. In these clinical studies, work intervals ranged from an intensity of 80–95% of heart rate reserve, and rest intervals ranged from 50–70% of heart rate reserve with a duration of 30–45 min per rehab session [7,8,9]. A recent meta-analysis which evaluated 16 studies (n = 969 patients) concluded that studies would benefit from being between moderate-to-vigorous and vigorous-intensity [10].

Hypertension, hyperlipidemia, diabetes, and obesity are cardiovascular risk factors that can be reduced with this type of exercise program [11,12], and which consequently have an influence on the reduction of chronic systemic inflammation [13], which has been shown to be an important risk factor for CVD [14]. The practice of regular exercise is associated with anti-inflammatory effects that are beneficial for health, mainly in patients with CVD, causing decreased levels of serum C-reactive protein [12], better cardiac output [15], stroke volume [15], vascular endothelial function [9], and changes in heart rate variability [16]. 

CR programs’ benefits are internationally consensual [1,2], but during the exercise, progressive physiological effects occur on the body temperature, heart rate variability (HRV), blood pressure, and cortical arousal, which have not been studied yet in CR programs. The real question is, what are the physiological differences between cardiac patients and healthy people during exercise, and is it possible to predict the appearance of the disease in people who are clinically healthy or who present an equivocal cardiac clinical condition?

Actually, new evaluation and control methods are applied to different sport areas such as performance, but also health. One of these is the analysis of the HRV as a tool to understand the autonomous nervous system status and response to different stimulus [17,18], facts directly related to heart and cardiovascular pathologies [19]. The analysis of HRV is based in the study of differences in milliseconds (ms) between RR waves of the electrocardiogram; then, using linear, frequency, or nonlinear analysis methods, we can analyze the autonomic nervous system response [20,21]. The other method is the use of thermography analysis, which allow us to study microcirculation abnormalities and capillarity disorders to prevent injuries and detect in early stages [22,23].

This case control study aims to analyze the physiological parameters of thermography, HRV, blood pressure, and cortical arousal in cardiac patients who belong to CR programs of HIIT and MICT, compared to healthy participants.

## 2. Materials and Methods

### 2.1. Participants

Two patients were recruited within the cardiology unit of the Hospital of Évora (Portugal). Two patients who had undergone a heart attack and were referred by their cardiologist to the cardiac rehabilitation (CR) phase III, two months after angioplasty and low-risk medical recommendations, were evaluated for inclusion in this case control study. The inclusion criteria were age 18–80 years, who had left ventricular ejection fraction ≥ 45%, and were New York Heart Association (NYHA) functional Class I, II, or III. In addition, patients were excluded from the study if the following criteria were met: severe exercise intolerance, uncontrolled arrhythmia, uncontrolled angina pectoris, severe kidney or lung diseases, musculoskeletal or neuromuscular conditions preventing exercise testing or training, and signs or symptoms of ischemia. The control group included two healthy participants without cardiovascular diseases. 

#### Randomization and Masking

This case control study had four participants, two HAP patients (both male, age 35 and 48, respectively) and two healthy controls (both male, age 38 and 46, respectively) who were randomly assigned in a 1:1:1:1 allocation ratio to one of four groups: cardiac HIIT (n = 1), cardiac MICT (n = 1), control HIIT (n = 1), and control MICT (n = 1) (Table 1). All groups are comparable in age and weight, and the two heart attack patients (HAP) were similar in the extent of coronary artery disease, coronary risk factors, type of coronary event, or left ventricular ejection fraction (Table 1).

### 2.2. Outcome Measures and Assessments

#### 2.2.1. Exercise Testing 

Initially, participants read and signed an informed consent form on the first visit, and the two HAP were submitted to a clinical evaluation performed by a cardiologist. A supervised graded exercise test to record volitional fatigue, risks, or symptoms of ischemia was performed on a treadmill, using the Bruce protocol, before the intervention. The test was done in non-fasting conditions and under medication. Electrocardiography was recorded continuously, and blood pressure was measured with an arm cuff every 3 min. 

#### 2.2.2. Thermography, Heart Rate Variability, and Cortical Arousal

On the second visit, each participant completed a standardized questionnaire including demographic data, medical history, medication use, family history of CVD, and smoking status; then, the peripheral vascular response was collected using a thermography system in two different moments: pre- and post-treadmill protocol. All thermal images were collected in compliance with the European Association of Thermology guidelines [24]. The thermograms of each participant were obtained in a room with a controlled and constant temperature of 20 °C and 40% humidity. Participants were in the test room 20 min prior to the data collection in order to acclimatize, and all the data collection occurred in the morning to control circadian rhythms [25]. To analyze the thermographic images, we divided the body in different sections: head, chest, abdomen, right arm, right hand, left arm, left forearm, and left hand. The analysis of the skin surface temperature was conducted by locating the middle point of each body section, and through a circle at the center of each dorsal and palmar hand (diameter 70 × 70 mm), following previous procedures [26].

The Heart Rate Variability (HRV) was measured by a H10 chest strap (Polar ©nc., Kempele, Finland) and recorded using a RS800CX monitor (Polar Inc., Kempele, Finland). This wireless device was placed below the participants’ chest muscles, allowing a reliable recording [27]; then, the Kubios HRV software (v. 3.3) [28] was used to pre-process and analyze the HRV data. A median filter was applied to correct possible artifacts. This filter allows the identification of RR intervals shorter/longer than 0.25 s, compared to the average of the previous beats. Correction replaces the identified artifacts with cubic spline interpolation. All HRV indices were extracted using the MATLAB Release 2019a (The MathWorks, Inc., Natick, MA, USA). Time-domain, frequency-domain, and non-linear measures were extracted. For this study, we only considered the time domain and non-linear domains. The following metrics were calculated:

- Time-Domain Analysis: (a) square root of differences between adjacent RR intervals (RMSSD);
- Non-linear analyses:©) non-linear metrics: the RR variability from heartbeat to short term Poincaré graph (width) (SD1), the RR variability from heartbeat to long-term Poincaré graph (length) (SD2), short-term fluctuation of the detrended fluctuation analysis (alpha-1), long-term fluctuation of the detrended fluctuation analysis (alpha-2), and the sample entropy (SampEn), which measures the regularity and complexity of a time series.

The cortical arousal was measured by the critical flicker fusion threshold (CFFT) by a Lafayette Instrument Flicker Fusion Control Unit model 12,021 (Lafayette, IN, USA), using standards protocols previously used [29]. Participants were familiarized with the procedure by performing practice trials before testing. The practice was before the basal sample, in line with previous studies [17]. Three ascending trials were carried out; in each one, time was quantified as the amount of time that a student took to detect the changes in the lights from the beginning of the test until the moment of pressing a button [30]. We used the critical flicker fusion threshold (CFFT) in this research since it has been widely used in different contexts, such as education, pharmacy, sports, military, and to evaluate cortical arousal and central fatigue [31,32,33,34,35,36].

Finally, the perception of fatigue was measured by a visual analogue scale (VSA) wherein the subjective fatigue was scaled to a 0–100 scale, 0 being no fatigue and 100 being an extreme fatigue following similar VSA [37].

### 2.3. Protocol and Experimental Procedures

**Figure 1:**
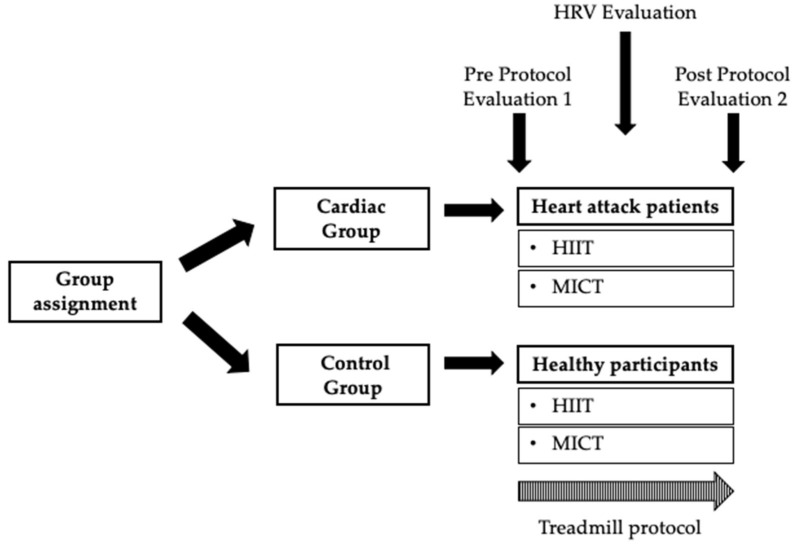
Summary of the present study protocol. HRV—Heart Rate Variability; HIIT—High-Intensity Interval Training; MICT—Moderate-intensity Continuous Training.

Regarding assessment procedures, participants had to rest for 15 min prior to baseline HRV collection in a sitting position, as recommended [38,39]. After 15 min at rest, 5 min of baseline was collected. Blood pressure, CNS fatigue, and cortical arousal were measured at the commencement and at the end of the session. The peripheral vascular response by thermography was collected at two different moments: pre- and post-treadmill protocols. The heart rate variability was collected: pre-, during, and post-treadmill protocols (Figure 1). Subsequently, participants performed an aleatory treadmill session of a CR program (HIIT and MICT), supervised by a physiologist (Figure 1). 

The assessments and data acquisition were performed by an external agent who was trained to do so, so that the researchers were totally blinded in the management of the data.

**Figure 2:**
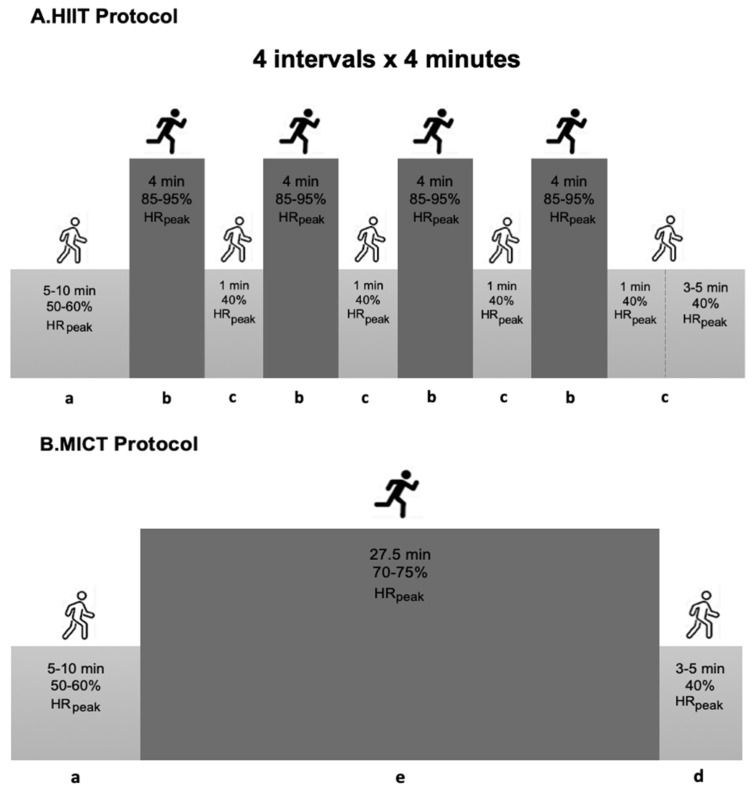
Summary of the exercise training protocol. HIIT—High-intensity Interval Training; MICT—Moderate-intensity Continuous Training; a—warm-up; b—interval bout of high-intensity exercise; c—one-minute recovery interval; d—cool-down; e—continuous bout of moderate-intensity exercise; min—minutes.

Training sessions on the treadmill were initiated with a 5–10 min warm-up at 50–60% peak Heart Rate (peakHR), and ended with 5 min of cool-down at 40% peakHR. The HIIT trial involved a total of 20 min at 85–95% peakHR, followed by a one-minute recovery interval at 40% peakHR, predicted with a supervised graded exercise test on a treadmill, using the Bruce protocol. During the high-intensity exercises, the participants were motivated to gradually increase their exercise intensity toward 15–17 on the Borg scale. The MICT protocol consisted of a continuous bout of moderate-intensity exercise to elicit 70–75% peakHR for 27.5 min, to equate the energy expenditure with the HIIT protocol (Figure 2).

As training intensity increased, the patients’ heart rate, rate of perceived exertion (Borg scale), and cardiac symptoms were also taken into consideration. 

### 2.4. Ethical Considerations

All work was conducted following the Declaration of Helsinki and registered at ClinicalTrials.gov (NCT03538119). Ethics approval was obtained from the University of Evora Ethics Committee (reference number: 17039). All participants signed a written informed consent before participating in this study.

## 3. Results

### 3.1. Thermography

**Figure 3:**
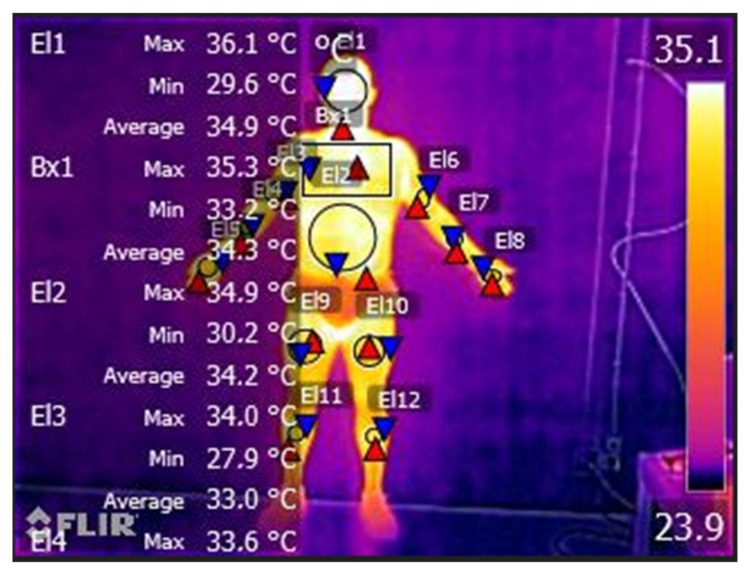
Temperature modification (°C) evaluated by thermography in Heart Attack Patients (HAP) and control participants in pre- and post- treadmill protocols (HIIT vs. MICT).

Before starting the protocols on the treadmill, the temperature was quite similar between the HAP and healthy participants’ groups. From pre- to post-protocols, there was always a decrease in temperature in all body variables evaluated in the study, except for the temperature of the right hand, where both HIIT groups increased temperature (temperature difference: 0.8 ± 0.5 °C in HAP vs. 1.0 ± 0 °C in control). In contrast, the MICT groups maintained the temperature from pre- to post-protocol. The same was not observed in the temperature of the left hand, which remained the same (Table 2, Figure 3).

The temperature difference in the chest was greater in patients with adverse cardiac events than in patients without events (temperature difference: 2.3 ± 1.2 °C in HIIT vs. 3.0 ± 1.6 °C in MICT). In the groups of healthy participants, the temperature remained practically the same. There was also a greater difference in temperature in the abdomen in the MICT group (temperature difference: 3.7 ± 1.8 °C in the HAP vs. 2.8 ± 0.0 °C in the control group) compared to the HIIT group (temperature difference: 1.5 ± 1.0 °C in the HAP vs. 1.0 ± 0.0 °C in control) (Table 2).

### 3.2. Heart Rate Variability

The stress index was higher in the HAP groups compared to the control groups. Those who did the HIIT protocol had higher Stress Index values from pre- exercise than those who did the MICT protocol, and from exercise to post-, the HAP in HIIT dropped slightly, while the HAP in MICT continued to rise sharply (Table 3). In addition, there was a higher decrease in the number of RR intervals in the HIIT in both groups (HAP: 210.5 ± 112.75 ms^2^ vs. control: 346 ± 0.00 ms^2^) compared to the MICT groups (HAP: 120.5 ± 74.2 ms^2^ vs. control: 81.7 ± 0.00 ms^2^). However, no significant interaction or main effects were observed in RMSSD (Table 3).

### 3.3. Central Nervous System Fatigue, Blood Pressure, and Cortical Arousal

Analyzing the fatigue of CNS in the different protocols performed, we verified that the continuous training presented greater fatigue of CNS for the HAP than in the control. However, the blood pressure difference was greater in patients with adverse cardiac events than in participants without events, and there were no differences in cortical arousal outcomes between the groups (Table 4).

## 4. Discussion

This research aimed to analyze the physiological parameters of thermography, HRV, blood pressure, and cortical arousal in cardiac patients who belong to CR programs of HIIT and MICT compared to healthy participants. Analyzing the fatigue perception of the different training conducted, we found that the MICT presented a higher fatigue perception for HAP than in control participants. It seems that the short rest interval allowed the HAP to have a lower fatigue perception, a fact in line with previous studies that also found higher motivation in interval training than in continuous training [40]. It is also important to note that HAP presented more than twice CNS fatigue in MICT than control participants, but HIIT had almost the same fatigue perception in HAP as control patients. We can see how MICT is more demanding for HAP, a fact that may explain the lower adherence to this training; in addition, whilst MICT is a training that is based on a traditional periodization, based on the sequencing of volume for an intensity during a certain period, which can make it less challenging, HIIT is identified more with a reverse periodization, based on an opposite paradigm—first the training intensity and then the volume [41]—and previous studies report that the level of adherence to reverse periodization was significantly greater than traditional training [42]; even so, it seems that the programs where greater adherence to CR programs is being verified are those that introduce virtual reality or video games [5].This result is important when practitioners have to design training for HAP since HIIT shows higher physiological adaptation [43]; furthermore, MICT in this population produces lower fatigability, a fact that would improve adherence to programs based on HIIT. In addition, independent of the training (HIIT or MICT), a hypotension response was evaluated, in fact, in line with previous studies, although recent research showed higher adaptations after HIIT protocols [44,45]. The same was also verified in patients with cardiac problems [46], which coincides with the results of our study regarding the fatigability of cardiac patients in mental and physical workouts. Still, no suggestions were made on the potential value of this method for the diagnosis or prognosis of cardiac disease.

Patients with hypertension or coronary disease tend to have low values for flicker fusion frequency. However, the patients without evidence of CVD also had values of the fusion frequency, and a positive correlation between flicker fusion frequency and resting systolic blood pressure have been found previously [46]. However, the patients without evidence of CVD also had values of the fusion frequency quite comparable with those for the cardiovascular patients, except for the group with malignant hypertension, but lower than for the normal people of equal age. Many types of pathology may depress flicker fusion frequency [47,48,49,50].

In the same regard, the present study showed that HIIT and MICT programs decreased systolic blood pressure in pre- to post-exercise. Mounting evidence demonstrates that participating in physical activity CR programs has been recommended to cardiac patients as an effective non-pharmacological approach to improving blood pressure [11,15,51].

There are studies that report the importance of heart rate variability in patients who have suffered heart attacks [52], as it seems that a reduced HRV is related to mortality after heart attack; thus, HRV can be a useful tool in risk stratification post-HAP [19,53]. Our findings showed that the HIIT protocol had improved the domains of HRV, including the number of RR intervals in HAP compared to MICT. In addition, some studies exposed that, compared with MICT, HIIT has good efficacy in improving cardiovascular fitness [10,43,45,54]. Furthermore, HIIT training appears to be a useful therapeutic intervention to improve the unbalanced autonomic function of HAP, and studies observed an increase in cardiac vagal activity after aerobic exercise programs [9,12,16]. However, our study observed no significant interaction or main effects in RMSSD. Regardless, the stress index of HRV was higher in the HAP groups compared to the control groups. The HIIT protocol had higher values from pre-exercise than those who did the MICT protocol, and from exercise to post-exercise, the HAP in HIIT dropped slightly, while the HAP in MICT continued to rise sharply. High values of stress index indicate reduced variability and high sympathetic cardiac activation. Similar exercise training programs have been provided. Some similar training programs showed different results, although some do not describe the loads applied in training [55,56,57,58]. Other authors report significant improvements in HRV using different training protocols [4,59]. Authors evaluated the cardiac autonomic response through HRV in women who performed a maximum incremental exercise; the results showed an abnormal autonomic modulation at rest, during, and after exercise [60,61,62], although other authors report that only two weeks of training with intensities above 75% can increase HRV [10].

Analyzing the thermography results, our study demonstrates that the body temperature difference in the chest was greater in patients with adverse cardiac events than in patients without events. In the groups of healthy participants, the temperature remained practically the same. Many authors propose diagnostic imaging as a means of detecting the risk of suffering from CVD [60,63,64]. Controlling inflammation in the carotid arteries may decrease the risk of CVD [63]. Using imaging as a diagnosis can prevent and help determine the cause of CVD [64]. Early signs of heart disease may be associated with increased or decreased peripheral blood flow. Thermography can play a key role in this diagnosis [65].

### Limitations of the Study and Future Perspectives

The main limitation of the present study is the low number of participants. Due to the specificity of the disease, namely, in the recovery phases (II on an outpatient basis or in phase III after medical discharge), it is still difficult to find participants to apply high-intensity exercise stimulus; thus, we decided to carry out a case study. Another limitation was the use of indirect measures of cortical arousal; an electroencephalography would more deeply explain all cortical responses in this population group. As perspectives for the future, we believe that this methodology is safe and can be beneficial in the recovery of patients who have suffered a heart attack (mainly in phase III of recovery after medical discharge), and can be a method of education or re-education towards healthier lifestyles. Therefore, we propose that this method be used in a larger sample of patients after a heart attack.

## 5. Conclusions

Finally, we concluded that both training protocols (HIIT and MICT) produced a similar thermographic response in both heart attack patients and control participants, showing in some body segments (such as chest, abdomen, right and left arm) lower temperatures in the heart attack patients. Regarding the autonomic response, heart attack patients presented higher sympathetic modulation in both trainings, showing that HIIT had higher sympathetic modulation than MICT; however, in the post evaluation, the HRV was equal between HIIT and MICT in heart attack patients. The MICT training produced higher subjective fatigue and a greater decrease in cortical arousal in heart attack patients than HIIT, contrary to that in control participants. No differences in systolic and diastolic blood pressure were found between HIIT and MICT training in heart attack patients; however, they presented higher systolic and lower diastolic blood pressure than control participants during both trainings.

## Figures and Tables

**Table 1:** Participant characteristics.

	HAP Group (n = 2)		Healthy Group (n = 2)	
	HIIT (n = 1)	MICT (n = 1)	HIIT (n = 1)	MICT (n = 1)
Demographics				
Age (years)	35	48	38	46
VO_2_peak (mL/kg/min)	30.7	30.4	33.3	32.7
Risk factors or comorbidities				
Body Mass index (kg/m^2^)	28.2	29.4	29.0	28.4
Waist Circumference (cm)	98.4	101.1	99.5	100.5
Left ventricular ejection fraction (%)	52	46	-	-
Diabetes mellitus	Y	Y	Y	Y
Hypertension	N	Y	N	N
Dyslipidemia	Y	Y	N	N
Active smoker	N	N	N	N
Family history of CVD	Y	Y	Y	N

CVD = cardiovascular diseases; HIIT = high-intensity interval training; MICT = moderate-intensity continuous training; VO_2_peak = maximal oxygen consumed; Y = Yes; N = No.

**Table 2:** Temperature in °C by thermography analysis in heart attack patients and control in the high-intensity interval training (HIIT) and moderate-intensity continuous training (MICT).

Variable	Group	Protocol	Pre	Post
Head (°C)	HAP	HIIT	34.1 ± 0.3	32.6 ± 0.8
		MICT	34.9 ± 1.3	33.4 ± 3.3
	Control	HIIT	34.4	32.7
		MICT	35.6	32.8
Chest (°C)	HAP	HIIT	34.6 ± 0.5	32.3 ± 1.8
		MICT	35.2 ± 1.6	32.2 ± 1.5
	Control	HIIT	34.7	33.5
		MICT	34.6	33.6
Abdomen (°C)	HAP	HIIT	34.0 ± 0.4	32.5 ± 1.6
		MICT	34.3 ± 2.5	30.6 ± 1.1
	Control	HIIT	34.3	33.3
		MICT	33.2	30.4
Right arm (°C)	HAP	HIIT	33.0 ± 0.1	31.2 ± 0.6
		MICT	34.7 ± 1.8	29.4 ± 0.4
	Control	HIIT	32.9	32.2
		MICT	33.5	31.9
Right forearm (°C)	HAP	HIIT	32.8 ± 0.4	31.1 ± 1.0
		MICT	33.8 ± 1.8	30.5 ± 0.5
	Control	HIIT	32.4	32.0
		MICT	34.0	32.3
Right hand (°C)	HAP	HIIT	31.9 ± 0.5	32.7 ± 0.5
		MICT	33.0 ± 2.0	33.2 ± 2.3
	Control	HIIT	32.3	33.3
		MICT	34.7	34.2
Left arm (°C)	HAP	HIIT	32.9 ± 0.6	30.5 ± 1.2
		MICT	34.3 ± 2.1	29.7 ± 0.8
	Control	HIIT	33.3	32.2
		MICT	33.5	30.3
Left forearm (°C)	HAP	HIIT	33.0 ± 0.8	30.5 ± 0.6
		MICT	33.6 ± 0.6	29.1 ± 0.0
	Control	HIIT	32.6	32.1
		MICT	33.7	31.9
Left hand (°C)	HAP	HIIT	32.0 ± 0.6	32.0 ± 0.7
		MICT	33.4 ± 1.1	33.1 ± 2.3
	Control	HIIT	32.8	32.8
		MICT	34.3	34.2

Data are presented as mean ± SD. HAP—Heart Attack Patients; CFFT—Critical Flicker Fusion Threshold; HIIT—High-intensity Interval Training; MICT—Moderate-intensity Continuous Training; °C—Celsius.

**Table 3:** Heart rate and heart rate variability parameters in heart attack patients (HAP) and control in high-intensity interval training (HIIT) and moderate-intensity continuous training (MICT).

Variable	Group	Protocol	Pre	Exercise	Post
Maximum heart rate (bpm)	HAP	HIIT	65.0 ± 7.1	137.012.7	96.0 ± 9.9
		MICT	78.0 ± 4.2	123.0 ± 24.0	95.5 ± 19.1
	Control	HIIT	84	170	97
		MICT	80	138	111
Average heart rate (bpm)	HAP	HIIT	69.5 ± 3.5	113.0 ± 9.9	87.0 ± 7.1
		MICT	62.5 ± 7.8	104.0 ± 18.4	87.0 ± 19.0
	Control	HIIT	78	133	89
		MICT	75	112	97
RMSSD (ms)	HAP	HIIT	27.9 ± 12.8	8.3 ± 1.7–19.6	10.9 ± 3.3
		MICT	23.4 ± 10.0	11.5 ± 6.8–11.9	9.5 ± 3.2
	Control	HIIT	25.3	10.2–15,1	16.3
		MICT	25	5.7–19.3	76.1
PNN50 (ms)	HAP	HIIT	9.5 ± 12.7	0.2 ± 0.0	0.4 ± 0.5
		MICT	4.3 ± 5.4	0.5 ± 0.6	0.5 ± 0.6
	Control	HIIT	4.2	0.8	0.7
		MICT	2.7	1.9	0.3
Stress Index	HAP	HIIT	12.4 ± 1.9	25.3 ± 6.6	21.3 ± 6.2
		MICT	16.3 ± 2.1	19.4 ± 2.9	35.7 ± 15.4
	Control	HIIT	10.9	15.3	16.1
		MICT	13	21	11.4
SD1 (ms)	HAP	HIIT	19.8 ± 9.0	5.4 ± 0.5	7,7 ± 2.3
		MICT	16.5 ± 7.1	8.1 ± 4.8	6.7 ± 2.3
	Control	HIIT	18	7.2	11.6
		MICT	17.7	13.2	53.6
SD2 (ms)	HAP	HIIT	41.0 ± 11.7	16.9 ± 6.1	27.5 ± 9.1
		MICT	26.5 ± 3.1	10.0 ± 4.8	11.2 ± 6.6
	Control	HIIT	52.9	27	40.5
		MICT	33.1	7.4	43.5
ApEn	HAP	HIIT	0.9 ± 0.0	1.0 ± 0.3	1.0 ± 0.0
		MICT	0.8 ± 0.1	1.4 ± 0.1	1.0 ± 0.0
	Control	HIIT	0.9	1.0	0.7
		MICT	1.0	1.4	1.0
SampEn	HAP	HIIT	1.8 ± 0.0	0.8 ± 0.4	1.2 ± 0.2
		MICT	1.6 ± 0.3	1.5 ± 0.2	1.7 ± 0.0
	Control	HIIT	1.2	0.9	0.8
		MICT	1.6	1.1	1.4

Data are presented as mean ± SD. HAP—Heart Attack Patients; CFFT—Critical Flicker Fusion Threshold; HIIT—High-intensity Interval Training; MICT—Moderate-intensity Continuous Training; RMSSD—Root Mean Square of Successive Differences; SD1—RR variability from heartbeat to short term Poincaré graph (width); SD2—RR variability from heartbeat to long-term Poincaré graph (length); ApEn—Approximate entropy; SampEn—Sample entropy; bpm—beats per minute.

**Table 4:** Fatigue of central nervous system, blood pressure and cortical arousal variables in heart attack patients and control in high-intensity interval training and moderate-intensity continuous training.

Variable	Group	Protocol	Pre	Post
Subjective fatigue scale (0–100)	HAP	HIIT	10.0 ± 0.0	67.5 ± 3.5
		MICT	10.0 ± 0.0	85.5 ± 3.5
	Control	HIIT	10	65
		MICT	10	40
Systolic blood pressure (mmHg)	HAP	HIIT	130.0 ± 26.9	121.0 ± 12.7
		MICT	132.5 ± 19.1	124.0 ± 18.4
	Control	HIIT	120	104
		MICT	124	138
Diastolic blood pressure (mmHg)	HAP	HIIT	80.0 ± 14.1	77.0 ± 2.8
		MICT	66.5 ± 13.4	73.0 ± 1.4
	Control	HIIT	72	83
		MICT	81	82
CFFT (hz)	HAP	HIIT	36.5 ± 7.7	37.9 ± 8.0
		MICT	38.2 ± 4.1	39.4 ± 4.5
	Control	HIIT	39.7	40.3
		MICT	39.7	41.5

Data are presented as mean ± SD. HAP—Heart Attack Patients; CFFT—Critical Flicker Fusion Threshold; HIIT—High-intensity Interval Training; MICT—Moderate-intensity Continuous Training.

## Data Availability

The data that support the findings of this study are available from the corresponding author, C.G., upon reasonable request.

## Abbreviations

CR	Cardiac rehabilitation
CVD	Cardiovascular diseases
CNS	Central nervous system
CFFT	Critical flicker fusion threshold
HAP	Heart attack patients
HRV	Heart rate variability
HR	Heart rate
HIIT	High-Intensity Interval Training
alpha-2	Long-term fluctuation of the detrended fluctuation analysis
ms	Milliseconds
MICT	Moderate-intensity Continuous Training
NYHA	New York Heart Association
peakHR	Peak Heart Rate
SampEn	Sample entropy
alpha-1	Short-term fluctuation of the detrended fluctuation analysis
RMSSD	Square root of differences between adjacent RR intervals
VSA	Visual analogue scale
WHO	World Health Organization
